# Differentially Expressed Genes Study Shown Potential for BCG Stimulation in Reducing the Severity of COVID-19

**DOI:** 10.1155/2022/1490408

**Published:** 2022-09-10

**Authors:** Irandi Putra Pratomo, Aryo Tedjo, Dimas R Noor, Wisnu Ananta Kusuma

**Affiliations:** ^1^Department of Pulmonology and Respiratory Medicine, Faculty of Medicine, Universitas Indonesia, Jakarta, Indonesia; ^2^COVID-19 Task Force-Pulmonology and Respiratory Medicine Unit, Universitas Indonesia University Hospital, Universitas Indonesia, Depok, Indonesia; ^3^Bioinformatics Core Facilities, Indonesian Medical Education and Research Institute, Faculty of Medicine, Universitas Indonesia, Jakarta, Indonesia; ^4^Drug Development Research Cluster, Indonesian Medical Education and Research Institute, Faculty of Medicine, Universitas Indonesia, Jakarta, Indonesia; ^5^Department of Medical Chemistry, Faculty of Medicine, Universitas Indonesia, Jakarta, Indonesia; ^6^Master's Programme Biomedical Sciences, Faculty of Medicine, Universitas Indonesia, DKI Jakarta, Indonesia; ^7^Human Cancer Research Center, Indonesian Medical Education and Research Institute, Faculty of Medicine, Universitas Indonesia, Jakarta, Indonesia; ^8^Department of Computer Science, Faculty of Mathematics and Natural Science, IPB University, Bogor, Indonesia; ^9^Tropical Biopharmaca Research Center, IPB University, Bogor, Indonesia

## Abstract

The incidence of COVID-19 infection and death is known to be lower in tuberculosis (TB) endemic countries than in nonendemic countries. The Bacillus Calmette-Guerin (BCG) vaccination, which is commonly administered in TB endemic countries, was previously reported to have a nonspecific protective effect against several infections, including COVID-19. In this study, we used a differentially expressed genes (DEG) approach to analyze the genes modulated by BCG vaccination and COVID-19 infection. The Gene Expression Omnibus (GEO) database was used to select a COVID-19 gene expression data set with GSE164805, GSE14408, and GSE58636, and DEG in each data set were identified using the GEO2R online tools and selected using the adjusted *p* value (padj) 0.05 criteria. The protein-protein interaction (PPI) network was constructed from DEGs with the same trend of expression (upregulation or downregulation) using STRING version 11. The PPI network was performed by using the highest confidence number (0.9). DEGs that have a high-trust network were collected and functional cluster analysis of biological processes from Gene Ontology (GO), pathway analysis from the Human KEGG pathway, and COVID-19-related gene analysis was carried out using the Enrichr database. We found that either BCG or tuberculin increased the expression of several genes related to hyperinflammation, such as CCL3, CCL4, CSF2, IL1B, and LTA. In severe COVID-19, these genes were downregulated. This leads to the hypothesis that revaccination may have a protective effect against the severity of COVID-19 by reducing the hyperinflammatory status.

## 1. Introduction

COVID-19 is a respiratory disease caused by SARS-CoV2 infection that has killed over 200,000 people worldwide [1]. At the beginning of pandemic times, developing countries were once predicted to face the highest number of deaths due to COVID-19. However, the mortality rate was found to be higher in developed countries such as the United States and Europe [2]. Madan et al. (2020) showed that COVID-19 incidence was lower in TB endemic areas compared to non-TB areas. Interestingly, the incidence of COVID-19 in nonendemic areas with high BCG coverage has a lower incidence of 4.3 per 100,000 compared to low BCG coverage of 46.6 per 100,000. However, the fatality rate of COVID-19 was lower in TB endemic areas (0 per 100 population), while in nonendemic areas the fatality rate was 1.42–1.43 per 100 population [3]. This suggests that BCG vaccinations may have a protective effect in TB endemic areas while the people that live in endemic areas have lower BCG vaccinations as well as assumptions about the role of TB's exposure to the COVID-19 incidence.

The mortality rate and severity of COVID-19 were found to be lower in *tuberculosis* (TB) endemic countries compared to some non-TB endemic countries such as Europe and the United States [4]. Recently, an epidemiological study conducted by Escobar et al. (2020) showed a significant relationship between Bacillus Calmette-Guerin (BCG) vaccination and a reduction in deaths from COVID-19. Furthermore, BCG vaccination is thought to have a protective effect against COVID-19 deaths, though more research into the mechanism is needed [5]. Recent evidence reported by Joy et al. (2021) using a meta-regression study from 160 countries showed that countries with BCG vaccination coverage above 70% had a lower incidence of COVID-19 infection than those of unvaccinated countries, except for the Middle East and North Africa [6].

Previously, Stensballe et al. (2005) reported that the BCG vaccine has a protective effect against the Respiratory Syncytial Virus (RSV), particularly for female infants.Reference [7]. In Indonesia, Wardhana et al. (2011) also reported that BCG vaccination in the elderly can reduce the risk of acute upper respiratory tract infection [8]. Another study conducted by Arts et al. (2010) reported the protection of the BCG vaccine against yellow fever. Reference [9]. The study suggested that the BCG vaccine can induce epigenetic clerical programming in humans and provide protection against unrelated viruses through increased IL-1 expression. Reference [9]. Through a study by Bell et al. (2021), it was shown that an increased expression of IL-1, along with IL-6 in the blood and tissue of COVID-19 patients, was not associated with severity [10]. Based on these two studies, BCG vaccination and COVID-19 severity may have a common expression pattern that may enhance the nonspecific antiviral protective effect. In this study, we used a computational approach based on differentially expressed genes (DEG) analysis from the Gene Expression Omnibus (GEO) database to identify differences or similarities in expression patterns between the BCG vaccine and COVID-19 infection.

## 2. Methods

### 2.1. Data Set

The COVID-19 gene expression data set (GSE164805) was selected from the GEO database (https://www.ncbi.nlm.nih.gov/geo/) based on the GPL26963 platform (Agilent-085982 Arraystar human lncRNA V5 microarray). GSE164805 consisted of 15 peripheral blood mononuclear cells (PBMCs) from patients with severe symptoms. As previously described by Wang et al. (2021) [11], the selection criteria include finding microarray studies on samples either of respondent PBMCs after BCG vaccination or TB antigen-stimulated PBMCs. The GSE14408 uses the GPL2700 Sentrix HumanRef-8 Expression BeadChip platform. The data collected from frozen PBMCs of 5 infant subjects who had previously received the BCG vaccine were thawed and stimulated with BCG or Purified Protein Derivative (PPD) of Tuberculin for 12 hours, with control of media without any stimulation (each group *n* = 5) [12]. The GSE58636 used the GPL10558 Illumina HumanHT-12 V4.0 expression BeadChip platform. The data set was collected from PBMCs from previously vaccinated adult subjects (group C, *n* = 13) and naive BCG (group A, *n* = 11), and compared their gene expression on the 7th day. Subjects in group C were then challenged with a standard dose of SSI BCG (*n* = 13). The PBMCs of subjects in the stimulated group C were then measured for their gene expression values on day 7, and compared their values with those of group C that were not stimulated with BCG (*n* = 12).

The data collected from frozen PBMCs from 5 infant subjects who had previously received BCG vaccine were thawed and stimulated for 12 hours with BCG or Purified Protein Derivative(PPD) of Tuberculin, with control of media alone, without stimulation for 12 hours (each group *n* = 5) [11]. The gene expression values of unstimulated PBMCs were compared with those of PPD and BCG stimulated. From GSE58636 on the GPL10558 Illumina HumanHT-12 V4.0 expression BeadChip platform, it was found that PBMCs from previously vaccinated adult subjects (group C, *n* = 13) and naive BCG (group A, *n* = 11) compared their gene expression on the 7th day. Subjects in group C were then challenged with a standard dose of SSI BCG (*n* = 13). The PBMCs of subjects in the stimulated group C were then measured for their gene expression values on day 7, and compared their values with those of group C that were not stimulated with BCG (*n* = 12).

### 2.2. Grouping

The subjects were grouped into several groups. The first group was patients with severe COVID-19 compared to healthy controls (1), the second group was adult subjects who had been vaccinated with BCG compared to BCG naive/unvaccinated adult subjects with BCG (2). The third and fourth groups were PBMC of infants who had been vaccinated with BCG and stimulated with BCG (3) and PPD (4), respectively, for 12 hours and compared to control. The fifth group was adult subjects who had been vaccinated with BCG, stimulated by a standard dose of BCG for 7 days compared to those that were unstimulated (5). The sixth group was adult subjects with BCG naive, stimulated by a standard dose of BCG for 7 days compared to those that were unstimulated (6). The summary of groups and gene expression that underwent changes, such as an increase or decrease in expression, are summarized in Table 1.

### 2.3. Data Processing and Identification of Differentially Expressed Genes

Differently expressed genes in each data set were identified using the online tools of GEO2R (https://www.ncbi.nlm.nih.gov/geo/geo2r/) provided by the NCBI. The DEG in each data set was selected using the adjusted *p* value (padj) 0.05 criteria. DEGs with the same expression pattern (upregulation or downregulation) in the three data sets were collected for PPI and enrichment analysis.

### 2.4. Protein-Protein Interaction Network Construction and Gene Function Enrichment Analysis

DEGs with the same expression trend in the three data sets were then mapped onto the STRING application version 11.0 (https://string-db.org/cgi/input.pl). A protein-protein interaction (PPI) network was created using the highest confidence (0.9). DEGs that have a high-trust network were collected and functional cluster analysis of biological processes from Gene Ontology (GO), pathway analysis from the Human KEGG pathway, and COVID-19 related gene analysis was carried out using Enrichr (https://amp.pharm.mssm.edu/Enrichr/).

## 3. Results

### 3.1. Stimulation of BCG and TB Antigen in PBMCs from the BCG Vaccinated Group Increased the Expression of Genes That Were Decreased in COVID-19

In this study, we investigated the potential protection of BCG vaccination against COVID-19. We selected data on gene expression results from PBMCs from several models and divided them into several groups. The grouping has been described previously in the methods.

In group 1, the expression of BIRC3, CCL3, CCL3L1, CCL4, CSF2, IL1B, LTA, LIF, and LTBR decreased, while the expression of CSF, CXCL1, CXCL2, CXCL8, LTBR, NFKBIA, OSM, and TNF was increased. In group 2, there was no significant difference between the expression of severe COVID-19 patients and adult subjects given the BCG vaccine except for the decreased OSM as shown in Table 1. In group 3, there was an increase in the expression of BIRC3, CCL3, CCL3L1, CCL4, CSF2, IL1B, and LTA, while the expression of CSF, CXCL1, CXCL2, CXCL8, NFKBIA, OSM, and TNF was also found to increase as in group 1. In group 4, there was also an increase in the expression of BIRC3, CCL3, CCL3L1, CCL4, CSF2, IL1B, LTA, and TNF, while the expression of LTBR, NFKBIA, and OSM was found to be insignificant. In groups 5 and 6, a similar pattern of expression changes was found. The expression of CCL3, CCL4, CSF2, CXCL, CSCL2, IL1B, LTA, OSM, and TNF was found to be increased, while the expression of BIRC3, CCL3L1, CSF3, CXCL8, LTBR, and NFKBIA was found to be not significant. Interestingly, an increased expression of CCL3, CCL4, CSF2, IL1B, and LTA in groups 5 and 6 was found, while the expression of these genes decreased in group 1.

### 3.2. PPI Analysis Showed That There Was a Centrality Equation between BCG Stimulations in the BCG Vaccinated Group

**Figure 1:**
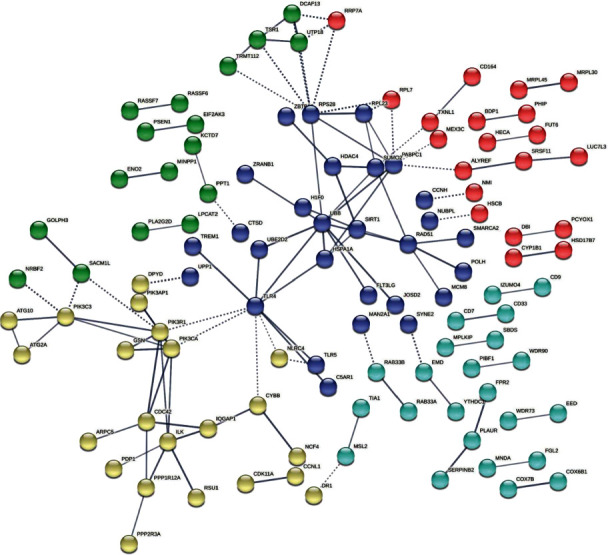
PPI analysis of genes expressed on PBMC of severe COVID-19 patients (Group 1) compared to PBMC of groups with single-dose BCG and naive vaccinated patients (Group 2). The PPI was created using the highest confidence score of 0.9.

**Figure 2:**
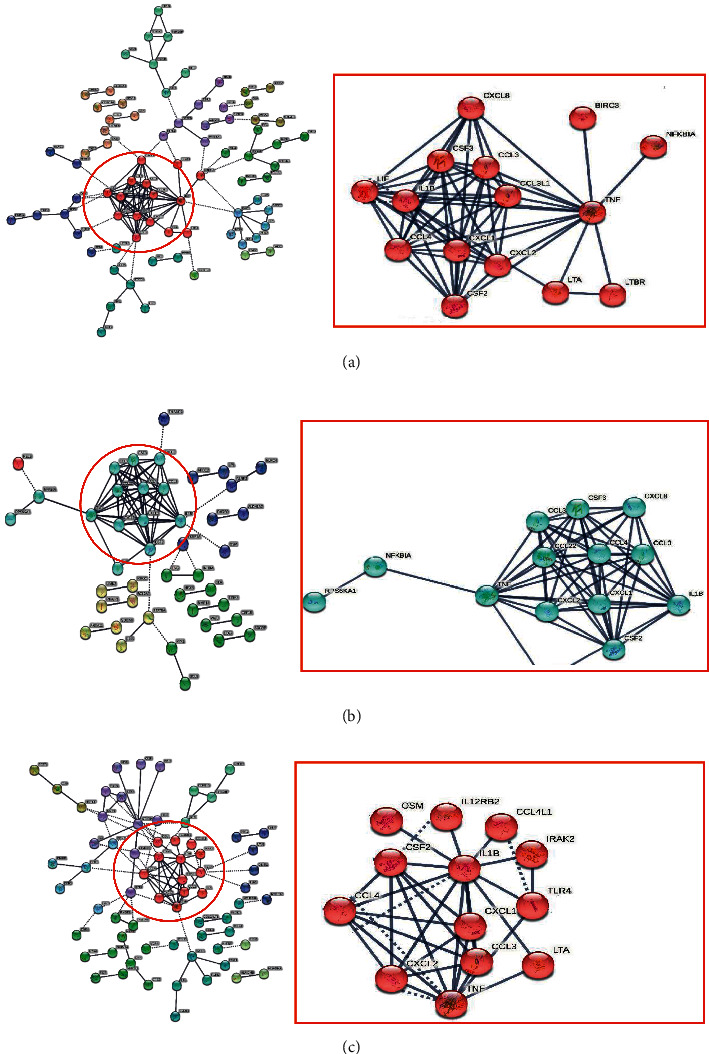
PPI analysis of genes expressed in severe COVID-19 to PBMC challenge with BCG or PPD. PPI of group 1 versus group 3 (a), group 1 versus group 4 (b), and group 1 versus group 5 (c). The PPI was created using the highest confidence score of 0.9. Annotation results show that different genes play a significant role in the severity of COVID-19.

In this study, we performed PPI to predict interactions of the expressed proteins of different genes from DEG analysis, particularly in groups 2, 3, 4, and 5, compared to group 1. There was no centrality found between groups 1 and 2, as shown in Figure 1. On the other hand, centrality was found when comparing groups 1 with 3, 4, and 5, as shown in Figure 2. The centrality consisting of CCL3, CCL4, CSF2, IL1B, CXCL1, CXCL2, CXCL8, and TNF was found in all groups, while the centrality between LTA and LTBR was found in group 3. In group 5, the centrality between LTA and OSM was found.

**Figure 3:**
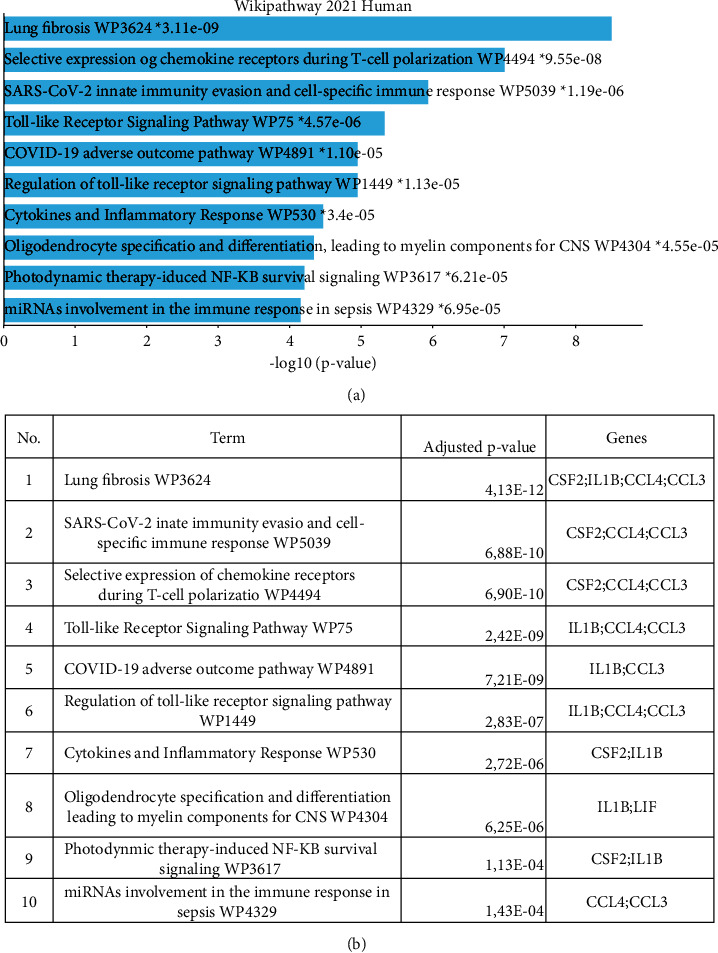
Annotations of genes contributed to COVID-19 severity. List of contributed pathways (a) and additional information about contributed pathways and list of genes (b).

To identify the biological processes associated with these genes, we continued by using annotation studies as well as identifying the most dominant pathways. The data is shown in Figure 3. Some of the genes that frequently appear are CCL3, CCL4, IL1B, and CSF2. These genes have been linked to pulmonary fibrosis, the selective expression of chemokine receptors required for T cell polarization, the SARS-CoV2 response in outwitting innate immune responses, and cell-specific immune responses. The involvement of CCL3, CCL4, and IL1B was observed in the toll-like receptor (TLR) pathway and the regulation of these pathways. COVID-19 outcomes were found to be adverse for CCL3 and IL1B. In addition, the roles of IL1B and CSF2 contribute to cytokine and inflammatory pathways. In addition, CCL3 and CCL4 are associated with microRNA involvement in sepsis-associated immune responses.

## 4. Discussion

Several studies have shown the protective effect of BCG vaccination against other risks of infection, such as RSV infection and yellow fever [7, 9]. Epidemiological studies conducted by Escobar et al. (2020) showed that BCG vaccination has a protective effect, although the mechanism remains unclear [5]. Therefore, we aimed to identify such a mechanism by using an analytical approach using DEG on the PBMC model, taken from several data sets, to get a clearer picture of the potential protection of BCG against COVID-19 infection.

According to the data set, BIRC3, CCL3, CCL3L1, CCL4, CSF2, IL1B, LTA, LIF, and LTBR expression was lower in severe COVID-19 patients (group 1) compared to healthy controls, while CSF, CXCL1, CXCL2, CXCL8, LTBR, NFKBIA, OSM, and TNF expression was higher. There was no significant change in the data set of severe COVID-19 patients when compared to BCG vaccination alone (group 2). In Figure 1, we show no centrality between the PPI. On the other hand, centrality that indicated strong interactions between significantly expressed genes began to be shown when PBMCs from in vitro experiments after BCG vaccination, either from a BCG or TB antigen group 3, as shown in Figure 2. This also showed that stimulation post-BCG vaccination increased expressions of BIRC3, CCL3, CCL3L1, CCL4, CSF2, IL1B, and LTA, which in contrast, decreased in severe COVID-19. Moreover, stimulation of a TB antigen, tuberculin, also showed an increased expression of BIRC3, CCL3, CCL3L1, CCL4, CSF2, IL1B, LTA, and TNF.

We also observed that PBMC of subjects that experienced stimulation from BCG revaccinations, also called BCG vaccine boosters, were found to have an increase in CCL3, CCL4, CSF2, CXCL, IL1B, LTA, OSM, and TNF, although BIRC3, CCL3L1, CSF3, CXCL8, LTBR, and NFKBIA were found to be not significant. An increased expression of CCL3, CCL4, CSF2, IL1B, and LTA was found in the revaccinated groups (groups 5 and 6), which in fact experienced decreased expression in severe COVID-19 patients. In Table 1 we show that in groups 3,4, 5, and 6 there was an increase in the expression of CCL3, CCL4, CSF2, IL1B, and LTA, whereas in group 1 there was a decrease. The centrality analysis of protein interactions showed that the expression of CCL3, CCL4, CSF2, and IL1B was observed most frequently.

Our findings of DEG were in line with that of Xu et al. (2020), who showed that the inflammatory response in PBMCs and lungs was reported to be different. According to those reports, the decreased expression of PBMCs actually indicated the paralysis of the immune system. Similar to what we found in this study, it was also reported that IL1B, CCL3, and CCL4 were lower in severe COVID patients (*n* = 19). [12] This, of course, can provide an illustration of the protective effect of BCG on the severity of COVID-19, which may be due to an increase in the expression of these genes, which in severe COVID-19 patients has decreased. BCG revaccination may be used as a strategy to prevent immune system paralysis and reduce the severity of COVID-19.

## 5. Conclusion

From this study, we found that BCG or PPD-stimulated increased expression of several genes related to inflammation, such as CCL3, CCL4, CSF2, IL1B, and LTA. In severe COVID-19, these genes were downregulated. Therefore, this emerging hypothesis of revaccination may have a protective effect against the severity of COVID-19 by reducing the hyperinflammatory status. This should be further confirmed and evaluated in PBMC.

## Figures and Tables

**Table 1:** Gene expression in PBMCs severe COVID-19 patients with PBMCs subject to BCG stimulation of PBMCs. Data that experienced upregulation were marked with green, downregulation with red, and not significant were marked with white lines.

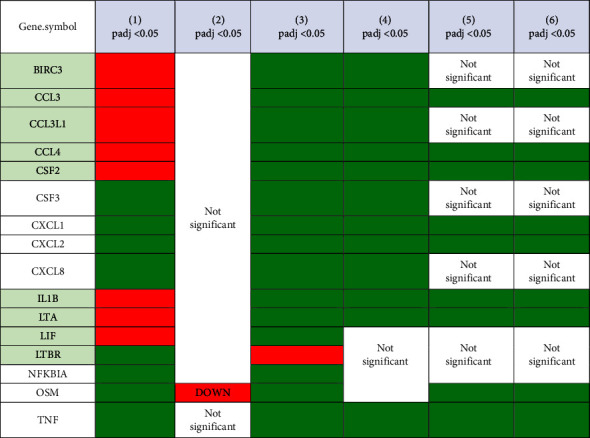

## Data Availability

Data are available on the database mentioned in this article, as it used secondary data from the Gene Omnibus Ontology (GEO) database.
